# Dentists and Dental Students

**Published:** 1872-09

**Authors:** H. L. Sage


### Dentists and Dental Students.

BY H. L. SAGE—MAY 29, 1872.

*Read Before the Connecticut State Dental Association.*

This subject, superficially surveyed, may seem of trivial importance, but it merits the serious consideration of those who are in the practice of taking dental students. The present status of the dental profession should lead such to see to it, that, so far as possible, the applicant is possessed of certain essential physical, moral, and mental qualities and endowments. The truth of this has become more and more apparent; and my convictions, founded upon observation and a little experience in this direction, have come to be stronger, and more perfectly defined.

Looking about also, and seeing the character, standing and ability of many who have assumed practice, one who is sensitive to the wants of the profession, will be seriously impressed by the danger there is, of accessions being made to its ranks, of individuals who will neither add to its dignity, worth, or usefulness. It is well-known that, of its members, a not inconsiderable number are a positive disgrace to it: or if not a positive disgrace, are dead weights, barring its progress.

Some, without principle, character, self-respect, or any just conception of what is expected or required of them by the profession, or the duties they owe to it, and society—without that laudable ambition or desire for the advancement of them selves and it in true worth, which characterizes the conscientious worker, are mere clogs and hinderances—robbing it, so far as they are concerned, of the respect which should attach to a calling, in itself noble and elevating.

Others, after having entered upon its duties, seem to rest satisfied, if they may but float along with the current, cast their nets, and draw in the fish of every kind.

There are, too, great "diversities of gifts," without "the same spirit." Hence, there is from the latter cause, little concert of action among the mass of dentists. But this does not apply to the majority of those who have associated themselves together in local, state, and national societies—a community of interests—for mutual improvement, and the perfecting of themselves and others.

But these comprise but a small proportion of the number who have assumed practice; and it is evident, that with this mixture of "good, bad and indifferent"—with the indifferent predominating, and the immoral in a ratio large enough to be painfully noticeable—the public (masses,) as at present educated upon the subject, have no just conception of the aim, object, and true work of the true dentist, so as in any just way to discriminate between the mere pretender, and the real possessor; of the requisites for a successful prosecution of the art, science and practice of dentistry; successful, as relating to the conservation of the organs with which he has to deal, and fulfillment, in the highest sense, of the duties devolving upon him.

Heinous and immoral conduct, such as drunkenness, lewdness, etc., of course can not be otherwise than too apparent for deception, and will, in time, bring merited disgrace upon the offender. Every profession is burdened with men of the "baser sort," and the dental is not exempt. But so far as incompetent and unqualified men, whether morally or otherwise, are concerned, the medical profession, by a *well-sustained* code of ethics, and the protection which the law affords, has more safeguards thrown about it, so that such can not so easily enter, or thrive within it. But in our own specialty, even without these means of protection, much may be done toward preventing accessions to its ranks, of those who will not add to its honor, efficiency, and usefulness.

It would seem that those who receive young men for purposes of instruction in dentistry, have, so far as they are concerned, in a great degree, a controlling influence in this direction. It should be insisted upon, that he who aspires to enter our laborious and self-sacrificing profession, should not only have a clear understanding of the character of the responsibilities to be assumed, but should, as before stated, be possessed of certain physical, moral and mental endowments and attainments, which, though at present too much ignored, are, in importance, becoming increasingly manifest to the earnest thinker, observer and co-laborer in this field of action.

It would be well, as this is the main object of this article, to consider some of the essential qualifications in the would be dental student and practitioner, the possession of which should be insisted upon by the private perceptor.

And the first, and by no means the least important, are the physical. "The health, strength, and vigor of the bodily organs and powers" should be viewed as they are—as extremely essential to the proper prosecution of the work which devolves upon the dentist.

Health is capital. One with a weak or impaired constitution, should seek some other sphere of usefulness, would he add length of days, happiness, and prosperity to his lot.

It may be assumed that, in no profession, are the pains and penalties resulting from the faithful practice thereof, more severe than in our own. Hence, the system should be capable of resisting the strain which is brought to bear upon it—otherwise, the premature decay and death of the bodily powers will be likely to follow. The consequences may be manifested in a moping, snail-like, or *sick-snail* movement, instead of the alacrity, buoyancy, and elasticity of motion which it is desirable that he should be enabled to put forth, naturally and involuntarily, because he feels like it. Most patients dislike a mope.

Aside from this, if physically incapacitated, he can not exhibit that cheerfulness and suavity of manner in his intercourse with patients, which is so desirable; while the lines of care upon his brow, or the pale and weary countenance, will oftentimes impress the patient, whether justly or not, with the idea that he is, from this cause, incompetent to properly perform his duties.

True, he may have learned the art of self-control to such an extent, as to assume a cheerfulness of demeanor, which does not reflect the usual workings of the impulses which would naturally spring from an impaired state of health, or a calmness and steadiness of exterior which shall say to the overtaxed nerves "peace, be still." Many there are, doubtless, in our profession, who possess this force of will, this soul power, which subdues and conquers. But the effort is often severe; and there are times, when such are betrayed into exhibitions of their physical weakness, by throwing off the mask which disguises it, and then the petulancy and impatience, will find expression in words and acts which will, perhaps, lose them the confidence and esteem of their patients.

He who in the midst of bodily suffering can at all times control his nerves, exercise this equanimity of temper, meet his patients with a smile, and agreeably and properly perform his duties, must possess somewhat of the grace of God, and by the force of will, reason, and understanding, have schooled himself by discipline to the task.

Indeed, he may prove himself a zealous and useful worker, notwithstanding those physical ailments. But had he chosen some avocation less trying, confining, and free from the contact with suffering patients—one in which more active, out-of-door exercise was inseparable from its prosecution, he might have realized the benefit in improved health, if not in longevity and a greater measure of worldly prosperity, if indeed he had not been capable of a greater degree of usefulness.

One should throw all his powers of mind and body into the profession which he adopts (aside from the duties which are not inconsistent therewith, and which are enjoined upon us as the first and highest), and the degree of his ambition, pride and excellence therein, will be varied or influenced by the state of his health.

If a sound body has not a controlling influence upon the clear and keen exercise of the mental faculties, it certainly has upon the working qualities of the physical, and upon pecuniary success and happiness. For he will, other things being equal, be able not only to accomplish more, but will have more to accomplish.

Also, the address of the individual, which Webster defines as "the manner of speaking to another," the exterior, the personal appearance, are matters of importance, and have much to do with success. But upon these I will not enlarge.

Not less important than the physical, are the moral qualifications. Indeed, the morals should be unexceptionable. No one lacking in this essential, can be safely employed in the practice of dentistry.

Who would knowingly trust a wife or daughter, needing professional services, with a man whose characteristics and tendencies were impure? This question becomes significant, when we consider that our profession has not a few dissolute, licentious, and unprincipled men in its ranks.

Clergymen and physicians, when impure in motives and in actions, wield an immense power for evil; and in a great degree, also, does the dentist, who, from the very nature of his position, must enjoy the confidence and esteem of his patients.

The clergyman is a teacher and preacher of morality, and, by the young especially, is looked upon as the exemplification of purity and Christian virtue. Next to, or equally with the minister, the family physician shares in the love and confidence of the household. Every well-regulated family has also their dentist; and to him is confided the care of the living organs which pertains to his specialty. From the nature of the trust reposed, which is something more than a reliance upon his professional ability, he should be a man above reproach; the soul of honor; pure in thought; whose words and actions will not bring a blush to the cheek of innocence; who will not take advantange of weakness; who has no conquests of which to boast; no insults to offer.

Many have an idea, that the dentist has peculiar opportunities for the exercise of the baser passions; and whether this be so in imagination, or reality, lady patients usually govern themselves accordingly, when placing themselves in his power; for instance, when anæsthetics are administered; and as it should be in such cases, by being accompanied by a friend. At other times they are not always exempt from insult, when in the hands of intemperate or unprincipled dentists. (It is surprising, but true, that this class will have many respectable people for their patients. The secret is, that their character and habits compel them to render their so-called services cheaply.)

Good morals, or what is better, religious principle, is also indispensable to the conscientious discharge of professional duty.

Otherwise, knowing, as the dentist does, that many patients are incapable of determining whether or not they have been justly dealt with, his selfishness, which, were it not so, would for the sake of the personal benefit lead to honorable service, drives him into temporary expedients for getting money, without caring whether the results of his operations are beneficial or lasting.

If his pride, or ambition to excel, or stand well with his professional brethren, does not overbalance his moral incompetency, woe be to the confiding patient? But this, with such, is very far from being the case. While a good man may be an indifferent dentist, from the want of natural gifts and acquirements in that direction, a bad man may be so from a want of common honesty, or an indifference to the good of those who seek his advice or aid.

A dentist of correct moral principles will carry out the spirit and intent of the code of ethics, not only so far as his obligations to his patients are concerned, but also, in all that attaches to, or is connected with the honorable standing, advancement and well-being of the profession. Were this much-to-be-desired qualification in universal possession of its members, there would be such a union of hearts and hands, that any desirable measure, or any much-needed reform could be effected, and the interests of the whole would be practically identical with, and inseparable from, those of every single individual.

Next, in regard to acquirements. While in a dental student, the possession of a liberal education is very desirable, a good English education is quite indispensable. A taste for, and habits of study, will add greatly to the success and usefulness of the individual, and will be essential to a correct understanding of much that comes within his province.

We can scarcely conceive of success in the true sense, without an amount of interest in his calling, which will impel him to enter, by study and research, into all its details, and so far as possible, the phenomena which have a bearing or influence, however remote, upon the organs with which he has to deal.

Neither should a young man be accepted as a student, without he signifies a willingness, and an intention, to avail himself of the benefits resulting from an attendance upon lectures at some dental college. In these days, it seems almost fool-hardy for one to think of entering upon practice without so doing.

True, many of our best, oldest, and most competent practitioners have not received these advantages, but they entered the profession many years ago, when dental education, as the term is now employed, was not thought so essential as at present. But were they to commence practice again, they would, without doubt, even at much personal sacrifice, if necessary, embrace the opportunity which is afforded. And the loss of these advantages is an ever-present souıce of regret. As it is, they have not reached their present position without effort, even though aided by a fair share of natural ability. But the importance of a dental education is too obvious to call for any extended argument to prove it.

This is the sum of the whole matter: with studious habits should be combined an aptness for the practical duties of the profession; with natural ability, judgment and skill, mental and moral culture; with physical stamina and endurance, honesty and uprightness of purpose; with the power of self-control, a nature, sympathetic and kind; with neatness of person and attire, order and system in the arrangement and execution of details; and if not naturally indowed with a well-balanced nature, a determination by well-directed personal effort to attain to as high a standard in these directions as possible.

Other planes of duty will then be reached, and other essentials realized.

